# Supplementary material for: Carrier-phonon decoupling in perovskite thermoelectrics via entropy engineering
Source: Nat Commun. 2024 Sep 3;15:7650. doi: 10.1038/s41467-024-52063-5 (PMC11369264; doi:10.1038/s41467-024-52063-5)
Supplement: Supplementary file 1 — Supplementary Information [file 41467_2024_52063_MOESM1_ESM.pdf]

Supplementary Information for

Carrier-phonon decoupling in perovskite thermoelectrics via entropy engineering

Yunpeng Zheng*, Qinghua Zhang*, Caijuan Shi*, Zhifang Zhou*, Yang Lu, Jian Han, Hetian Chen, Yunpeng Ma, Yujun Zhang, Changpeng Lin, Wei Xu, Weigang Ma, Qian Li, Yueyang Yang, Bin Wei, Bingbing Yang, Mingchu Zou, Wenyu Zhang, Chang Liu, Lvye Dou, Dongliang Yang, Jin-Le Lan, Di Yi, Xing Zhang, Lin Gu, Ce-Wen Nan and Yuan-Hua Lin†

†Corresponding author:

Prof. Yuan-Hua Lin E-mail: linyh@tsinghua.edu.cn

Table of Contents

Supplementary Notes 3

Supplementary Figures 8

Supplementary Tables..38

Supplementary References..49

Supplementary Notes

Calculation of the mean radius $\bar{r}$ and the size disorder parameter δ

To quantify the lattice distortion caused by atomic radius disorder, the mean radius $\bar{r}$ and the size disorder parameter δ were calculated[12].

$$\delta = \sqrt{\sum_{i=1}^{N} c_i \left(1 - \frac{r_i}{\bar{r}}\right)^2} \qquad \text{(Supplementary Equation 1)}$$

$$\bar{r} = \sum_{i=1}^{N} c_i r_i \qquad \text{(Supplementary Equation 2)}$$

Where N, c_i, r_i are the number of elements at the crystal site, the molar percentage of the *i* th element, the effective ionic Shannon radius of the *i* th element.

Calculation of effective mass and Lorentz Factor by SPB model

To describe the transport behaviors of thermoelectrics, the single parabolic band model (SPB model) was used. By using SPB model under certain electron-phonon interaction, the DOS effective mass (m_d^*), Lorentz Factor, Pisarenko curve could be deduced from the Hall measurement of mobility (μ_H) and carrier concentration (n). The correlations between Seebeck (S), n, m_d^*, Lorentz Factor (L), and temperature (T) were expressed as follows[3]:

$$S = \pm \frac{k_B}{e}\left[\eta_F - \frac{\left(r+\frac{5}{2}\right)F_{r+3/2}(\eta_F)}{\left(r+\frac{3}{2}\right)F_{r+1/2}(\eta_F)}\right] \qquad \text{(Supplementary Equation 3)}$$

$$n = \frac{4}{\sqrt{\pi}}\left(\frac{2\pi m_d^* k_B T}{h^2}\right)^{\frac{3}{2}} F_{1/2}(\eta_F) \qquad \text{(Supplementary Equation 4)}$$

$$F_{\mathrm{i}}(\eta_{\mathrm{F}})=\int_0^{\infty}\frac{x^{\mathrm{i}}\mathrm{d}x}{1+\exp(x-\eta_{\mathrm{F}})} \quad \text{(Supplementary Equation 5)}$$

$$\eta_{\mathrm{F}}=\frac{E_{\mathrm{F}}}{k_{\mathrm{B}}T} \quad \text{(Supplementary Equation 6)}$$

$$L=\frac{k_{\mathrm{B}}^2}{e^2}\frac{\left(r+\frac{3}{2}\right)\left(r+\frac{7}{2}\right)F_{r+1/2}(\eta_{\mathrm{F}})F_{r+5/2}(\eta_{\mathrm{F}})-\left(r+\frac{5}{2}\right)^2F^2{}_{r+3/2}(\eta_{\mathrm{F}})}{\left(r+\frac{3}{2}\right)^2F^2{}_{r+1/2}(\eta_{\mathrm{F}})} \quad \text{(Supplementary Equation 7)}$$

where $F_i(\eta_{\mathrm{F}})$ is the Fermi-Dirac integral, η_{F} the reduced Fermi level, r the scattering factor, h the Planck constant, k_{B} the Boltzmann constant, e the elemental charge.

When the electrons are dominantly scattered by phonons, the $r = -1/2$, so:

$$S=\pm\frac{k_{\mathrm{B}}}{e}\left[\eta_{\mathrm{F}}-\frac{2F_1(\eta_{\mathrm{F}})}{F_0(\eta_{\mathrm{F}})}\right] \quad \text{(Supplementary Equation 8)}$$

$$L=\frac{k_{\mathrm{B}}^2}{e^2}\frac{3F_0(\eta_{\mathrm{F}})F_2(\eta_{\mathrm{F}})-4F^2{}_1(\eta_{\mathrm{F}})}{F^2{}_0(\eta_{\mathrm{F}})} \quad \text{(Supplementary Equation 9)}$$

Calculation of weighted mobility and electronic quality factor

To understand the intrinsic electrical performance and the potential of the materials, the weighted mobility and the electronic quality factor which are n-independent were calculated[45].

$$\mu_{\mathrm{W}}=\frac{3h^3\sigma}{8\pi e(2m_e k_{\mathrm{B}}T)^{3/2}}\left\{\frac{\exp\left(\frac{|S|}{k_{\mathrm{B}}/e}-2\right)}{1+\exp\left[-5\left(\frac{|S|}{k_{\mathrm{B}}/e}-1\right)\right]}+\frac{\frac{3}{\pi^2}\frac{|S|}{k_{\mathrm{B}}/e}}{1+\exp\left[5\left(\frac{|S|}{k_{\mathrm{B}}/e}-1\right)\right]}\right\}$$

(Supplementary Equation 10)

$$B_{\mathrm{E}} = \frac{S^2\sigma}{\frac{S_r^2 \exp(2-S_r)}{1+\exp[-5(S_r-1)]} + \frac{S_r\pi^2/3}{1+\exp[5(S_r-1)]}} \quad \text{(Supplementary Equation 11)}$$

$$S_r = \frac{|S|}{k_{\mathrm{B}}} \quad \text{(Supplementary Equation 12)}$$

Calculation of minimum thermal conductivity

The amorphous limit by Cahill model could be calculated according to[6]:

$$\kappa_{min} = \left(\frac{\pi}{6}\right)^{\frac{1}{3}} k_B n^{\frac{2}{3}} \sum_i v_i \left(\frac{T}{\Theta_{\mathrm{i}}}\right)^2 \int_0^{\Theta_i/T} \frac{x^3 e^x}{(e^x-1)^2} dx \quad \text{(Supplementary Equation 13)}$$

Where k_{B}, n, v_i, T are Boltzmann constant, the number density of atoms, sound velocity, temperature in Kelvin, and $\Theta_i = v_i \left(\frac{h}{2\pi k_B}\right)(6\pi^2 n)^{\frac{1}{3}}$.

Calculation of bond length and observed tolerance factors from PDF spectra

For SLTO, SBLTO, SBCLTO, SBCPLTO, the unchanged elements were La (20 atomic %), so the average length of La-O bonds were used to represent the length of A-O bonds for calculating the tolerance factor. The average length (La-O) and length (Ti-O) were calculated by the integral average calculation from the *G* (*r*)-*r* curves.

$$length = \frac{\int_a^b r \cdot 4\pi r^2 G(r)\mathrm{d}r}{\int_a^b 4\pi r^2 G(r)\mathrm{d}r} \quad \text{(Supplementary Equation 14)}$$

a and *b* are the start and end point of the peaks.

$$t_{\mathrm{obs}} = \frac{length(A-O)}{\sqrt{2}\, length(B-O)} \quad \text{(Supplementary Equation 15)}$$

Calculation of phonon mean free path l_{p}

The longitudinal and transverse sound velocities v_l and v_t were measured by ultrasonic pulse-echo method, and the average sound velocity v_a was calculated by

$$v_a = \left[\frac{1}{3}\left(\frac{1}{v_l^3} + \frac{2}{v_t^3}\right)\right]^{-\frac{1}{3}} \quad \text{(Supplementary Equation 16)}$$

The κ_L and C_V were simultaneously measured by LFA, and the phonon mean free path l_p was calculated by

$$\kappa_L = \frac{1}{3} C_V v_a l_p \quad \text{(Supplementary Equation 17)}$$

Calculation of carrier mean free path l_c

In the pseudo-cubic thin films, for simplification, the inertial effective mass $m_I^* \approx m_b^*$, and the band effective mass was calculated from the density of states effective mass m_d^* and the degeneracy N_V by:

$$m_d^* = N_V^{\frac{2}{3}} m_b^* \quad \text{(Supplementary Equation 18)}$$

and the degeneracy of pseudo-cubic perovskites was six by approximating[7].

The longitudinal sound velocity was from the bulks, and the density was calculated using molar mass and lattice parameters. And the C_l was calculated by

$$C_l = \rho v_l^2 \quad \text{(Supplementary Equation 19)}$$

The deformation potential Ξ_{def} was calculated by[8]

$$B_E = \frac{2N_V \hbar C_l}{3\pi m_I^* \Xi_{def}^2}\left(\frac{k_B}{e}\right)^2 \quad \text{(Supplementary Equation 20)}$$

And the carrier mean free path was calculated by[9,10]

$$l_c = \frac{h\mu_H}{2e}\left(\frac{3n}{\pi N_V}\right)^{\frac{1}{3}} \quad \text{(Supplementary Equation 21)}$$

Calculation of normalized Ti displacement

The lengths of Ti-O_a and O_a-O_b (marked in Fig. 4a) in different octahedrons of entropy engineered thin films were measured from the STEM-ABF images and were analyzed to get the normalized Ti displacement:

$$d_{Ti} = \frac{length(C-Ti)}{length(C-O_b)} = \frac{\frac{1}{2}length(O_a-O_b)-length(Ti-O_a)}{\frac{1}{2}length(O_a-O_b)} \qquad \text{(Supplementary Equation 22)}$$

The average values and the standard deviations were also calculated.

Supplementary Figures

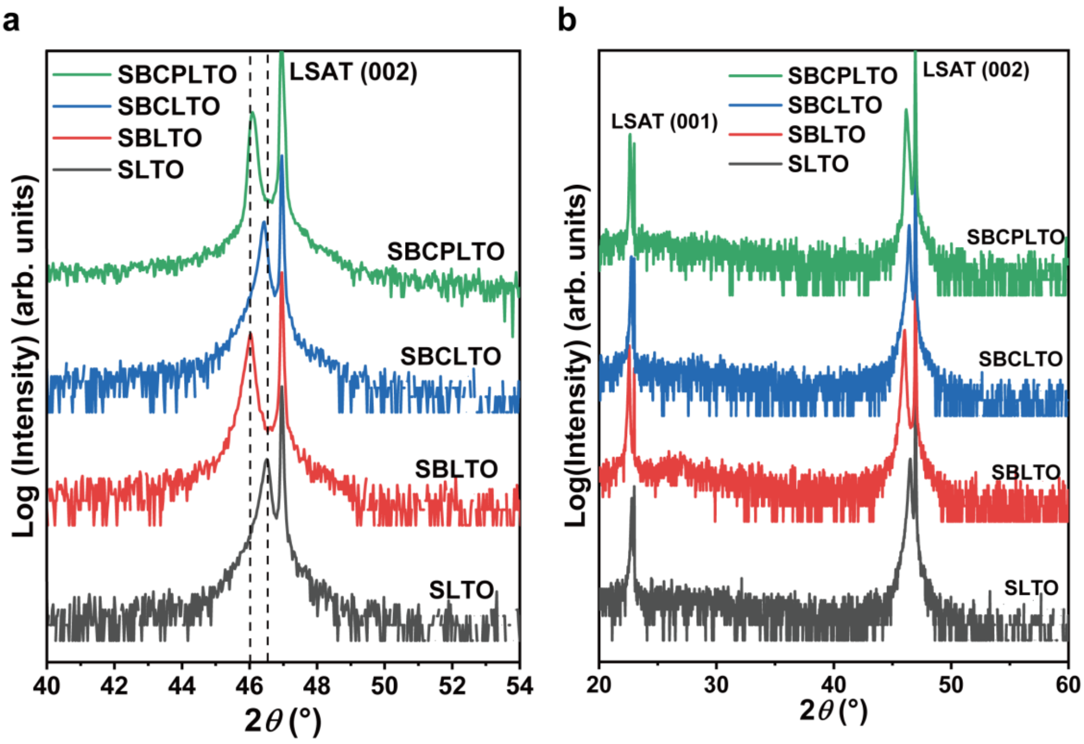

Supplementary Fig. 1 | XRD. a. The out-of-plane XRD patterns of (002) peaks of entropy engineered films and LSAT (001) substrates. The dashed lines were drawn as guidance for eyes to the peak shifts after introduction of elements of different radii. **b.** XRD patterns showing (001) and (002) peaks of LSAT (001) substrates and entropy engineered thin films, SLTO, SBLTO, SBCLTO, SBCPLTO.

Notes for Supplementary Fig. 1:

The films were all epitaxially grown, and the high-quality growth was reflected by the sharp and strong (002) peaks of thin films.

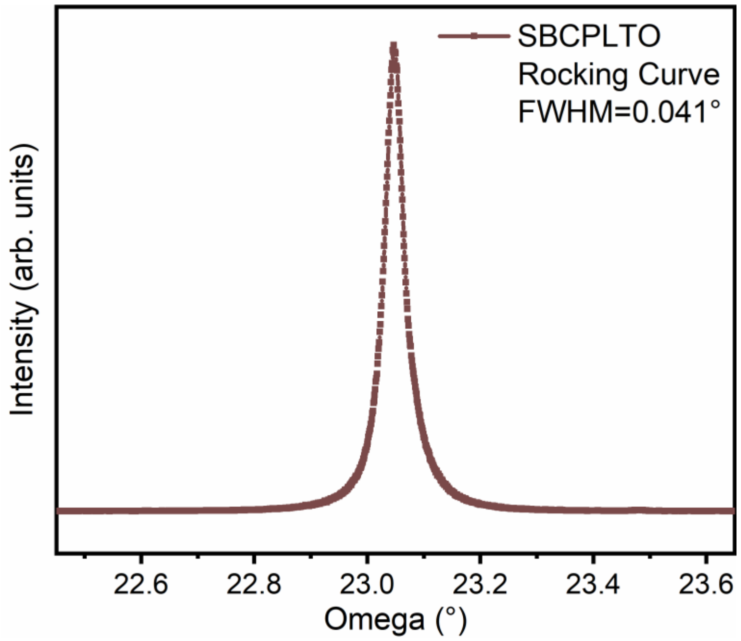

Supplementary Fig. 2 | Rocking curve. The Omega scan of SBCPLTO (002) diffraction peak.

Notes for Supplementary Fig. 2:

The full width half maximum (FWHM) of out-of-plane (002) peak of SBCPLTO was 0.041°, which reflected high quality of the epitaxial growth in this high-entropy thin film.

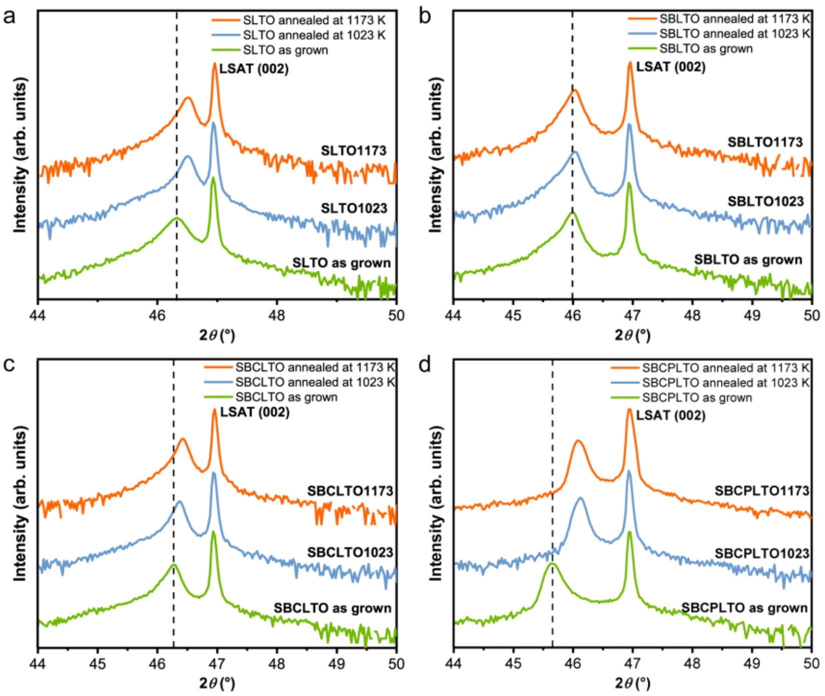

Supplementary Fig. 3 | XRD. a.-d. The (002) XRD peaks of SLTO (**a**), SBLTO (**b**), SBCLTO (**c**), SBCPLTO (**d**) thin films annealed at different temperatures.

Notes for Supplementary Fig. 3:

The (002) XRD peaks of as-grown thin films, 1023 K annealing and 1173 K annealing thin films were shown, and the annealed samples displayed single epitaxial phases. The peaks of films shifted towards higher angles after annealing. In these conductors (annealed films) with itinerant electrons, the Ti^{4+} did not change valence, and the chemical contraction was owing to the oxygen vacancies created[11,12]. The chemical contraction could reflect the formation of oxygen vacancies. It could be seen that the annealing was effective, and the effects of 1023 K and 1173 K annealing were similar.

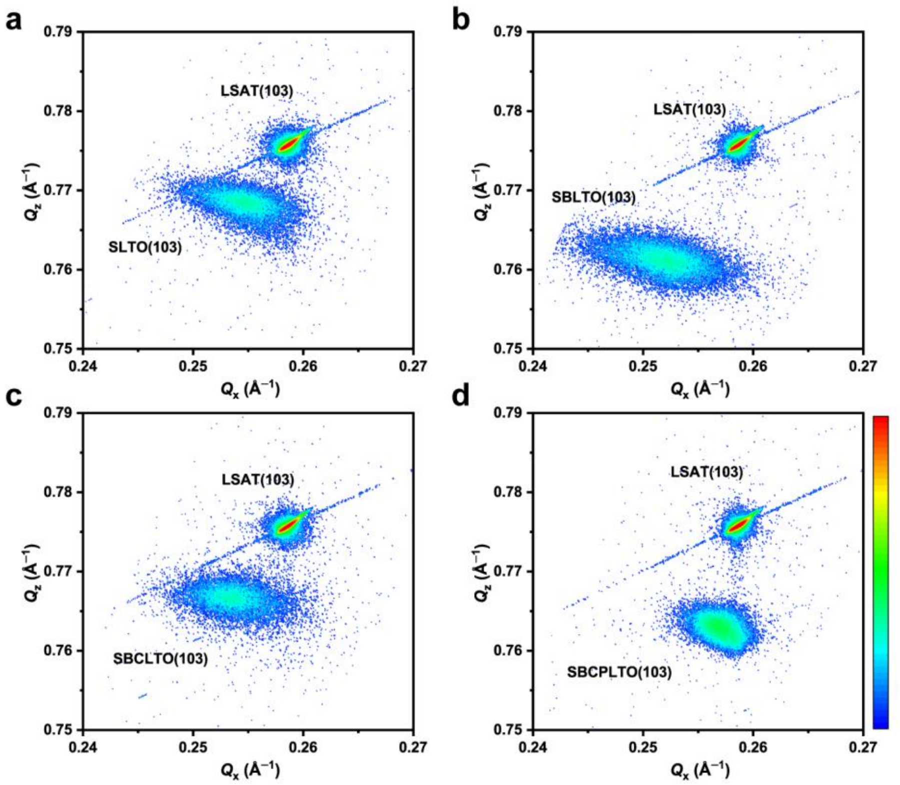

Supplementary Fig. 4 | RSM. a.-d. The RSM images of (103) peaks of the entropy engineered films by X-ray diffraction (XRD).

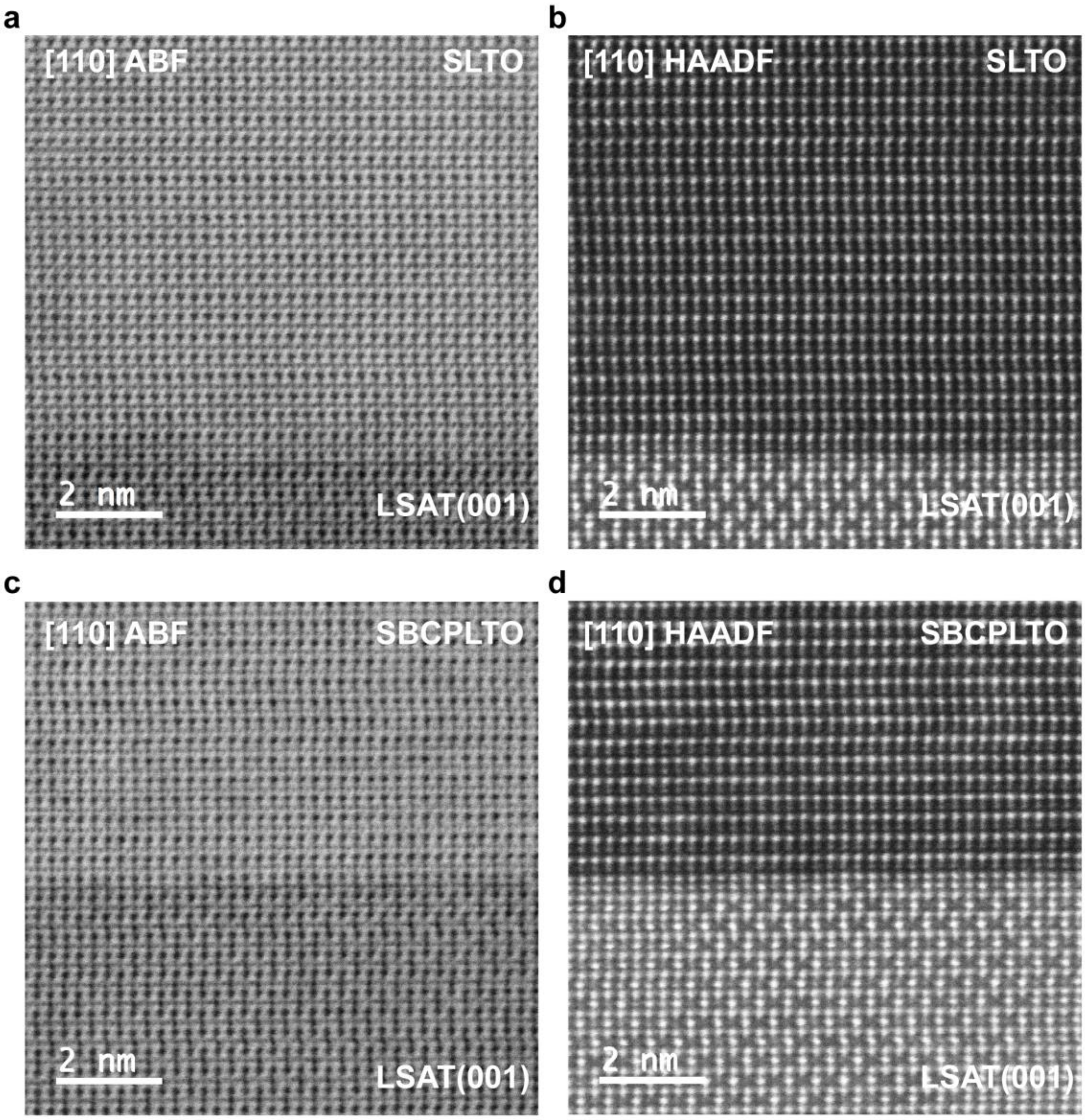

Supplementary Fig. 5 | STEM along [110] direction in atomic resolution. a. ABF of SLTO. **b.** HAADF of SLTO. **c.** ABF of SBCPLTO. **d.** HAADF of SBCPLTO.

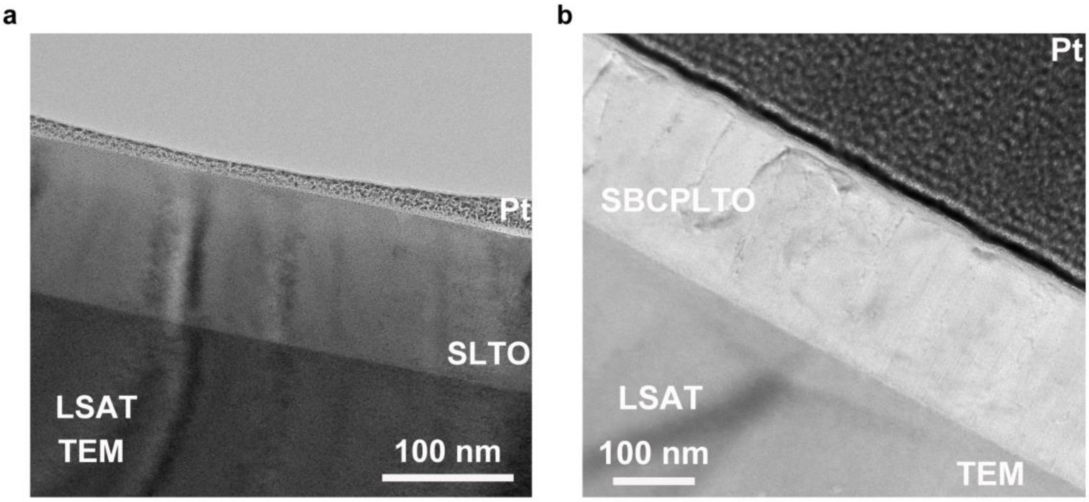

Supplementary Fig. 6 | Cross-sectional TEM images. a. SLTO. **b.** SBCPLTO.

Notes for Supplementary Fig. 6:

In the TEM images, clear interfaces and the cross-sectional morphology of thin films were shown. The low- and high-entropy thin films were of high quality. Since the obvious influence of grain boundaries on carrier[13] and phonon[14] transport, the high quality epitaxially grown perovskite thin films with the optimized orientation (001)[11] could be an ideal platform to show the effects of entropy engineering on transport behaviors, excluding extrinsic effects. The weakened carrier-phonon transport was found and explored.

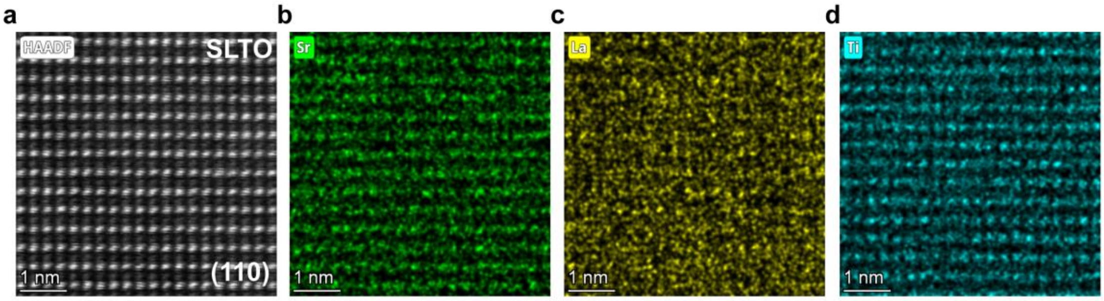

Supplementary Fig. 7 | The elemental distribution of SLTO in atomic resolution. a. HAADF along [110]. **b.-d.** The STEM-EDS mapping of Sr (**c**), La (**c**), Ti (**d**). The elements distributed uniformly in atomic resolution in SLTO.

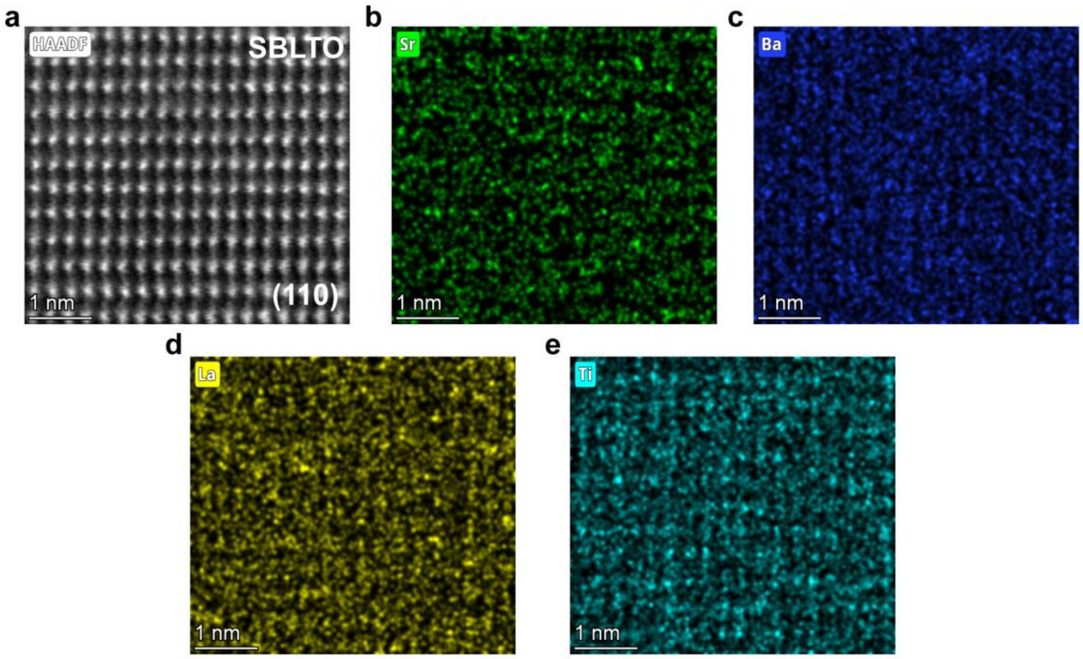

Supplementary Fig. 8 | The elemental distribution of SBLTO in atomic resolution. a. HAADF along [110]. **b.-e.** The STEM-EDS mapping of Sr (**b**), Ba (**c**), La (**d**), Ti (**e**). The elements distributed uniformly in atomic resolution in SBLTO.

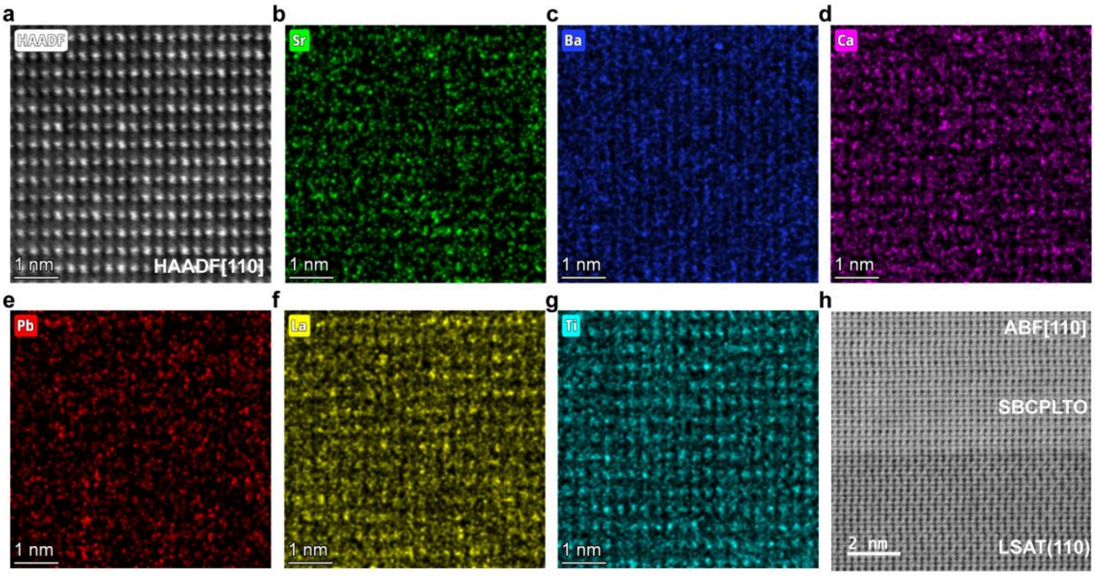

Supplementary Fig. 9 | STEM characterization providing information on structure, elemental distribution and interfacial epitaxy of SBCPLTO. a. Atomic resolution HAADF of SBCPLTO along (110) direction. **b.-g.** Corresponding EDS mapping. **h.** ABF image of the interface of SBCPLTO.

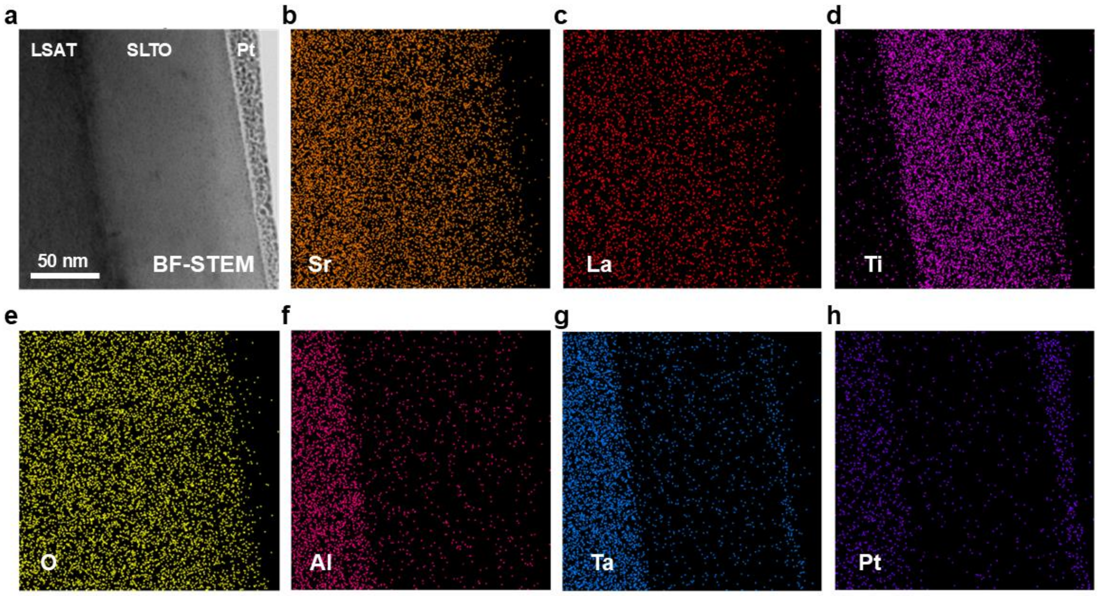

Supplementary Fig. 10 | The elemental distribution of SLTO at mesoscale level by STEM-EDS. The elements distributed uniformly at mesoscale level in SLTO.

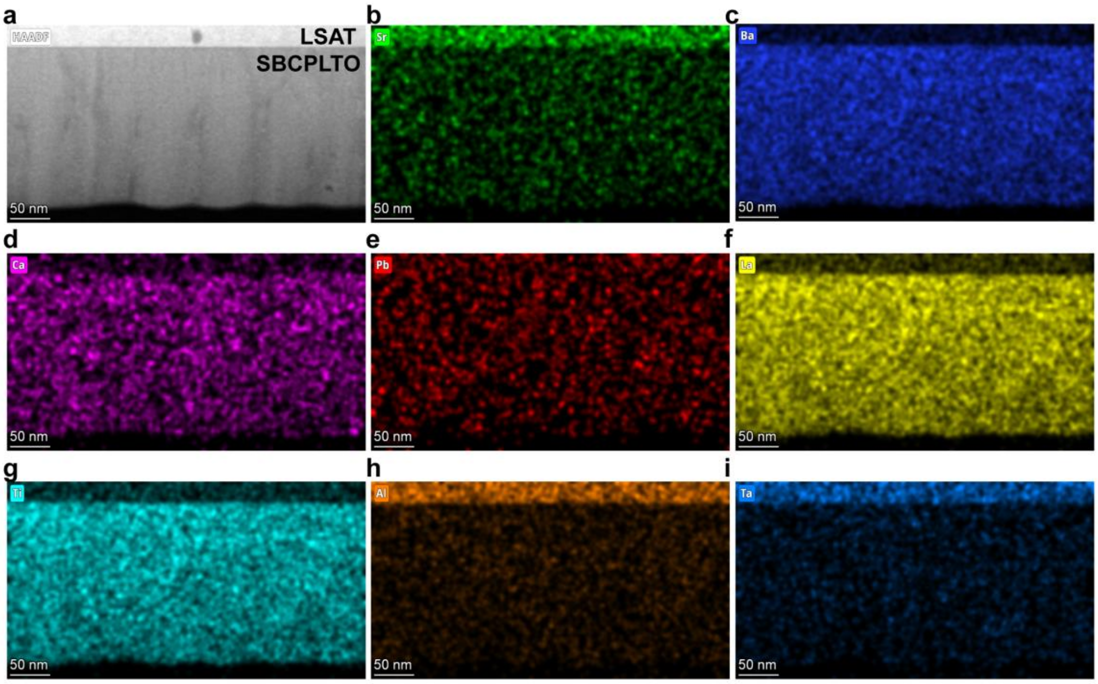

Supplementary Fig. 11 | The elemental distribution of SBCPLTO at mesoscale level by STEM-EDS. The elements distributed uniformly at mesoscale level in SBCPLTO.

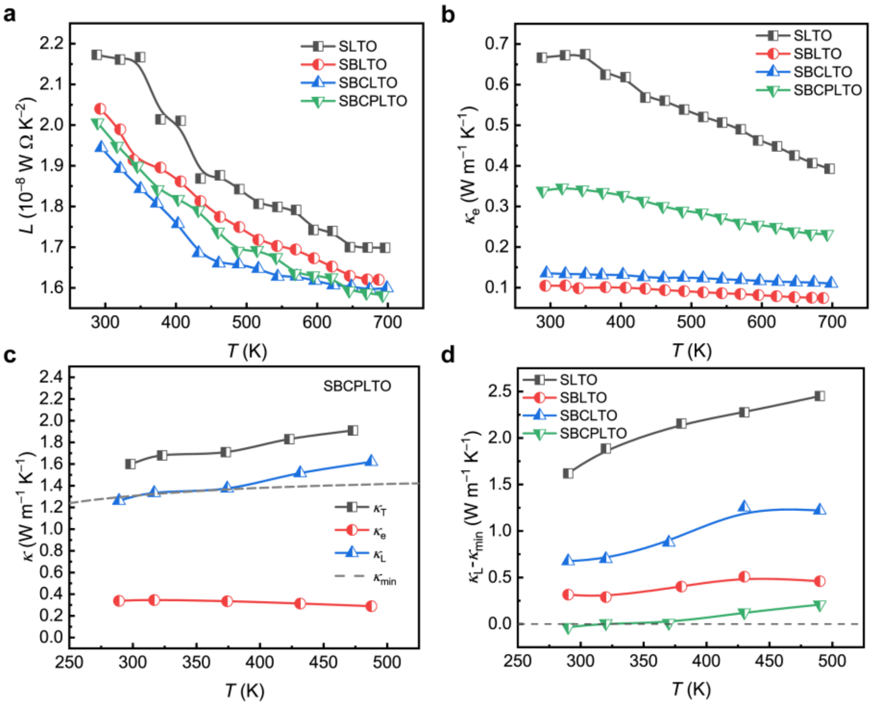

Supplementary Fig. 12 | The analysis of thermal transport. a. The calculated temperature dependent Lorentz factor of entropy engineered thin films. **b.** The temperature dependent electronic thermal conductivity of entropy engineered thin films. **c.** The detailed thermal transport contribution from lattice and electrons of SBCPLTO. The dashed line is the calculated amorphous limit thermal conductivity of SBCPLTO. **d.** The temperature dependent κ_L-κ_{min} of entropy engineered thin films.

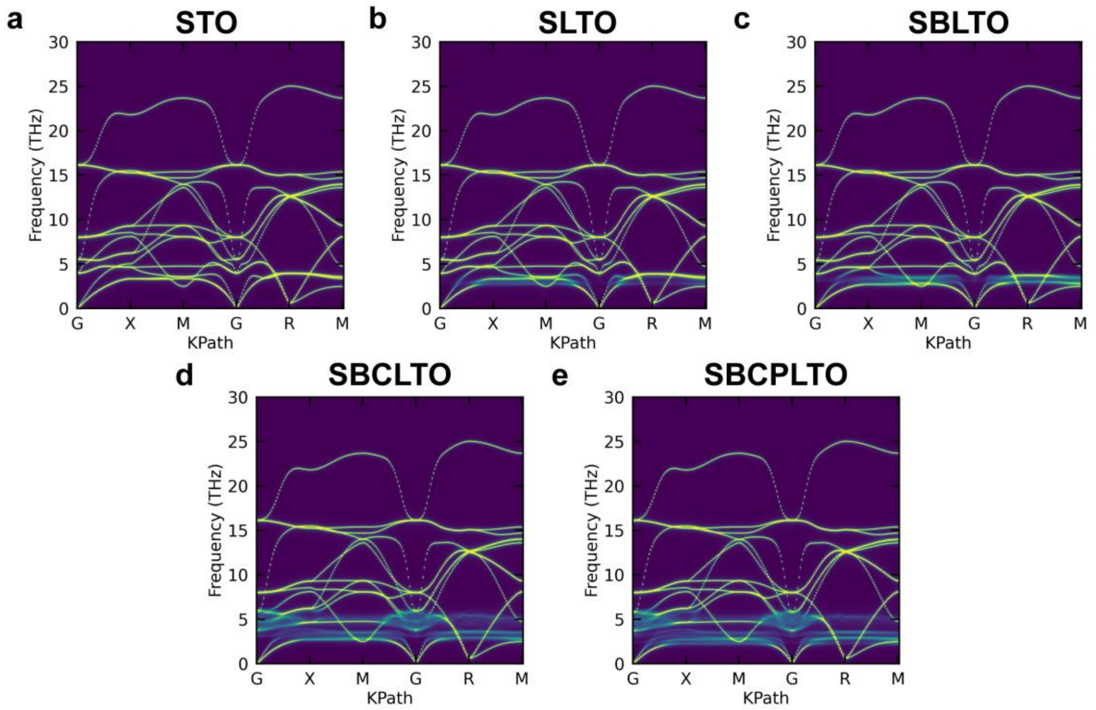

Supplementary Fig. 13 | The phonon dispersion calculation results. a. STO. **b.** SLTO. **c.** SBLTO. **d.** SBCLTO. **e.** SBCPLTO.

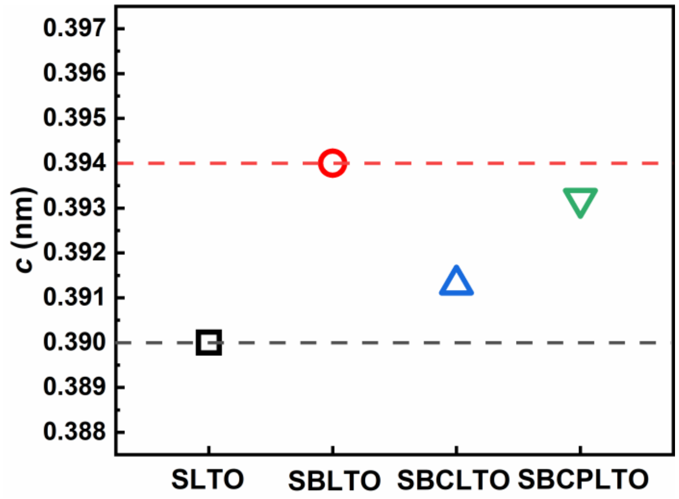

Supplementary Fig. 14 | The out-of-plane lattice parameter *c* of entropy engineered annealed thin films. The dashed lines mark the position and serve as guides for eyes.

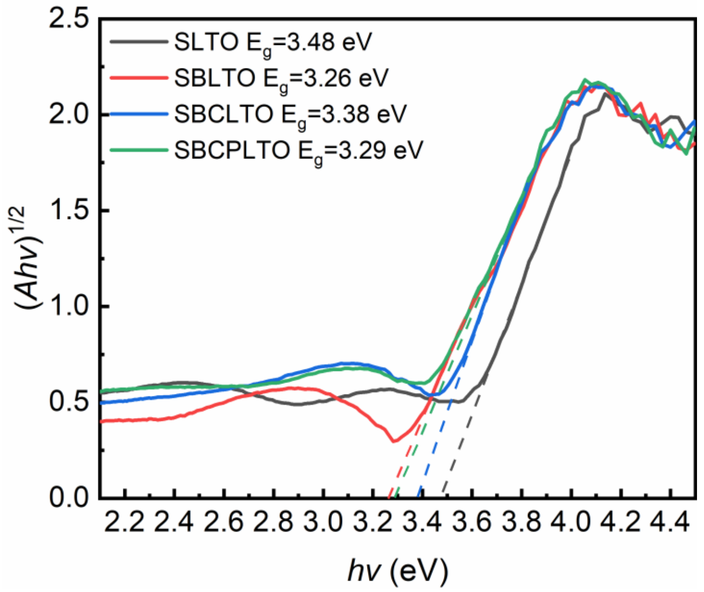

Supplementary Fig. 15 | The Band gap measurement on entropy engineered thin films by UV-vis spectrophotometer.

Notes for Supplementary Fig. 15:

The STO-based oxides have an indirect band gap of ~3.3 *e*V[7,15], so the UV-vis spectrophotometer was used to measure the wide band gap, and the $(Ahv)^{1/2}$-hv (in *e*V) curves for indirect band gap were plotted and fitted to get the band gaps. The band gaps were reversely correlated to the lattice parameters, which is common in materials with similar crystal and band structures. The distortion of TiO_6 might contribute to the red shift in SBLTO[16], and the Pb 6*p* might also slightly narrow the band gap in SBCPLTO.

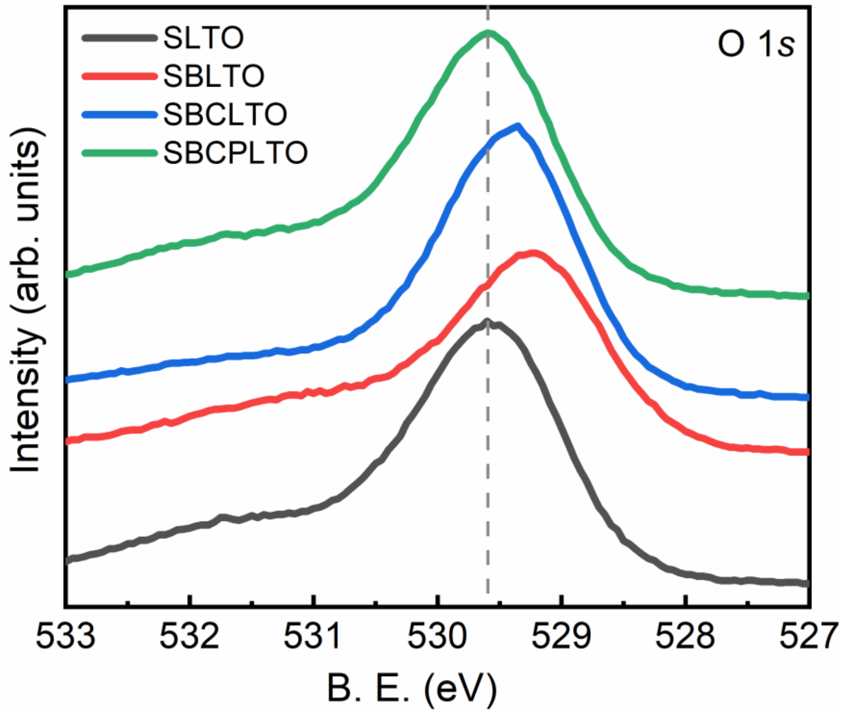

Supplementary Fig. 16 | The XPS characterization of O 1*s* on entropy engineered thin films. The dashed line marks the peak position and serve as guides for eyes.

Notes for Supplementary Fig. 16:

The O 1*s* XPS peaks showed similar peak shifts with Ti 2*p*, for the binding energy was decided by the chemical environment, TiO_6 octahedrons.

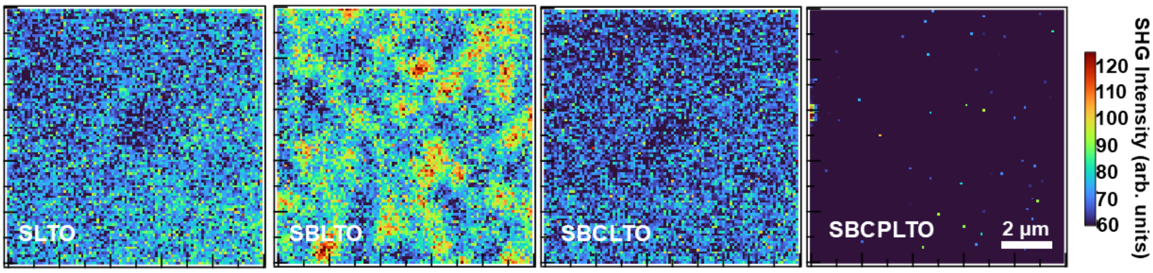

Supplementary Fig. 17 | The in-plane SHG mapping of entropy engineered thin films.

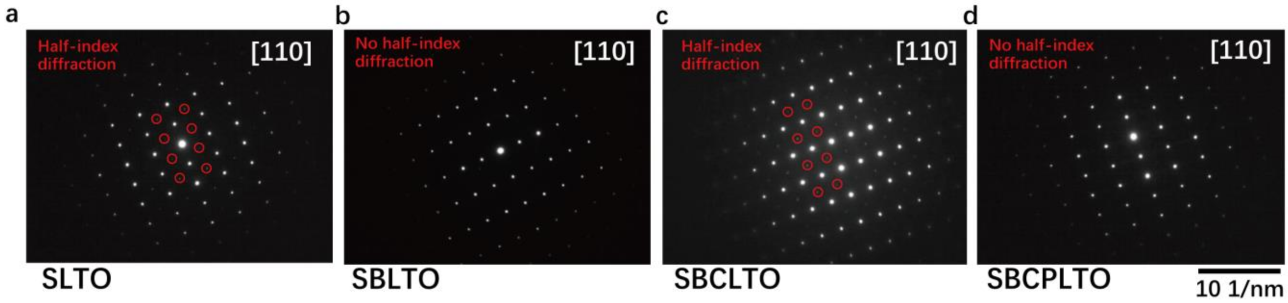

Supplementary Fig. 18 | The SAED images of entropy engineered thin films along [110] direction. a. SLTO. **b.** SBLTO. **c.** SBCLTO. **d.** SBCPLTO. According to the presence and the absence of the half-index diffraction, it is easy to tell the presence and absence of octahedron tilting in the samples. Red circles highlight the half-index diffractions.

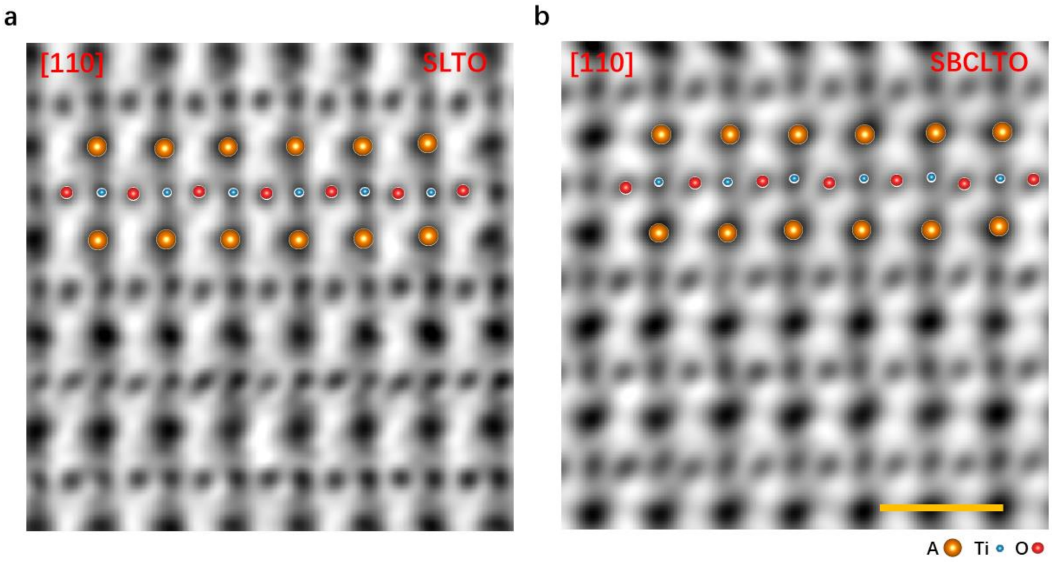

Supplementary Fig. 19 | The filtered STEM-ABF images in atomic resolution to view directly the tilting of TiO_6 octahedrons. a. SLTO. **b.** SBCLTO. The orange scale bar denotes 5 Å. The orange, red, and blue spheres represent A-site atoms, O, and Ti, and the overlapped spheres were omitted.

Notes for Supplementary Fig. 19:

The SAED half-index diffraction could only tell the presence and absence of octahedron tilting in the samples, while in the STEM-ABF images, the more tilted TiO_6 octahedrons in SBCLTO than those in SLTO were directly observed.

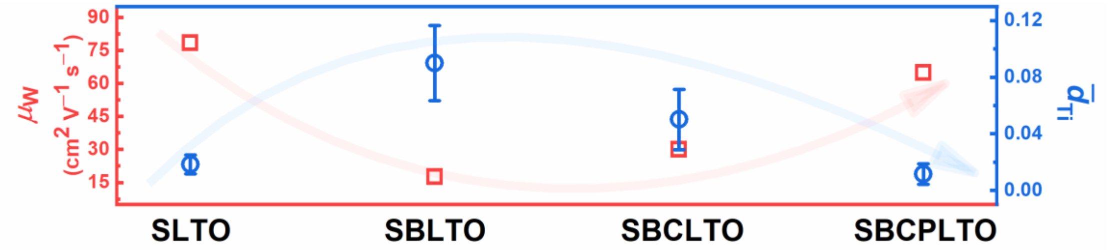

Supplementary Fig. 20 | The relation between room temperature weighted mobility (μ_W) and average normalized Ti displacement ($\bar{d}_{Ti}$) of SLTO, SBLTO, SBCLTO, and SBCPLTO with increased entropy. The error bar is the standard deviation δ_d. The blue and the red arrows are the guides to show the trends of weighted mobility and displacement.

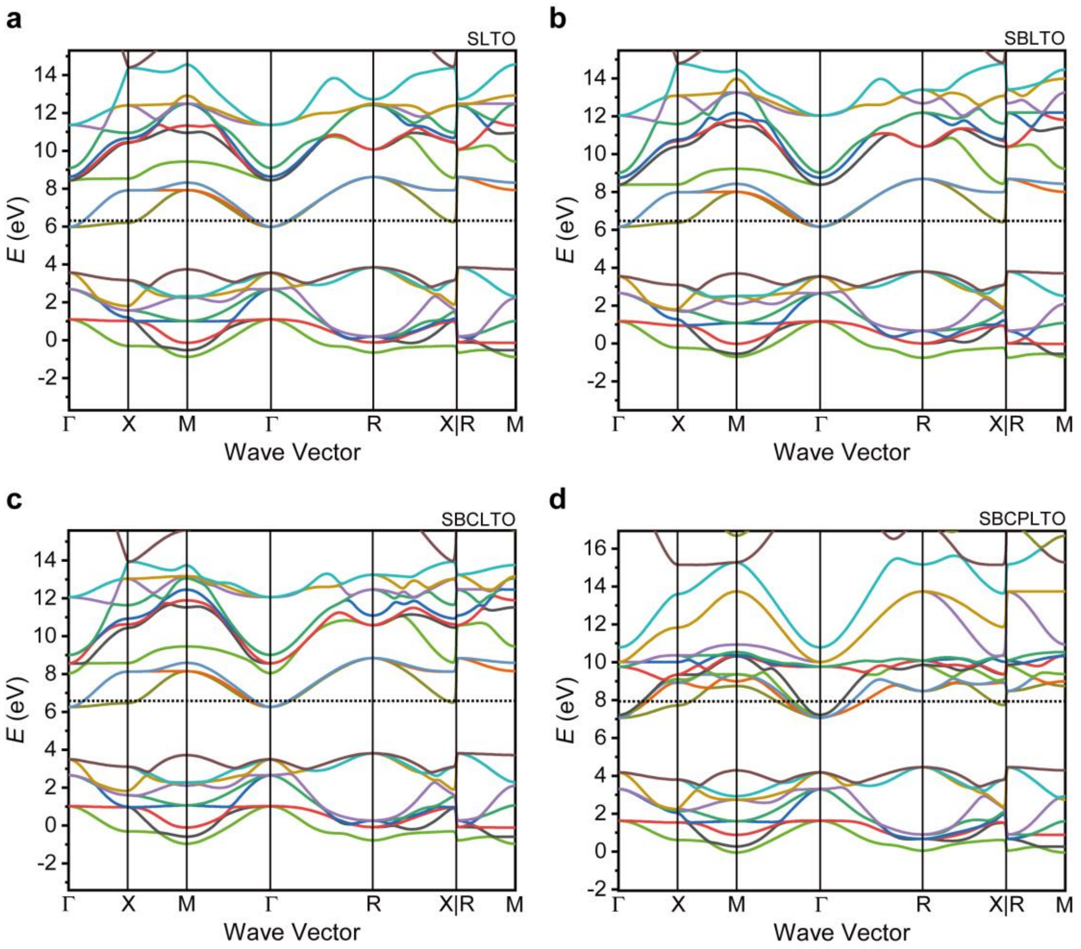

Supplementary Fig. 21 | The energy band dispersion calculation results. a. SLTO. **b.** SBLTO. **c.** SBCLTO. **d.** SBCPLTO. The Fermi level is marked by dashed line.

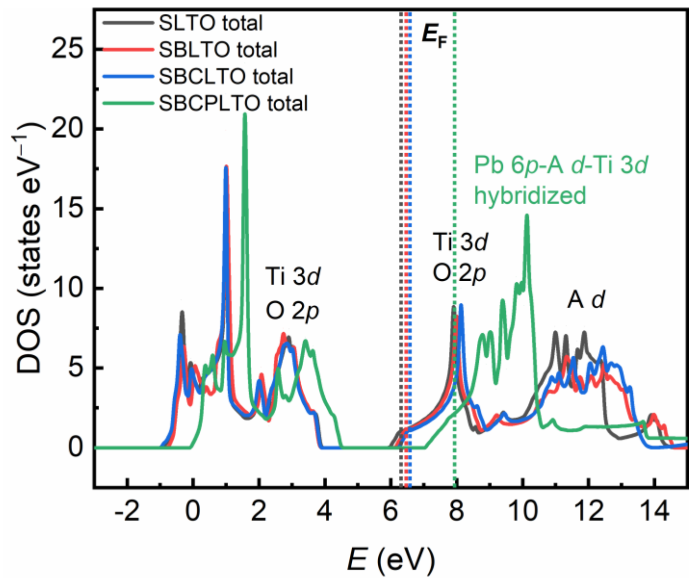

Supplementary Fig. 22 | The total DOS of entropy engineered perovskites. The main orbital contribution is marked in the figure, and the Fermi level is marked by dashed line in the same color with curves of corresponding composition. The atom of site A is Sr, La for SLTO; Sr, Ba, La for SBLTO; Sr, Ba, Ca, La for SBCLTO; Sr, Ba, Ca, Pb, La for SBCPLTO.

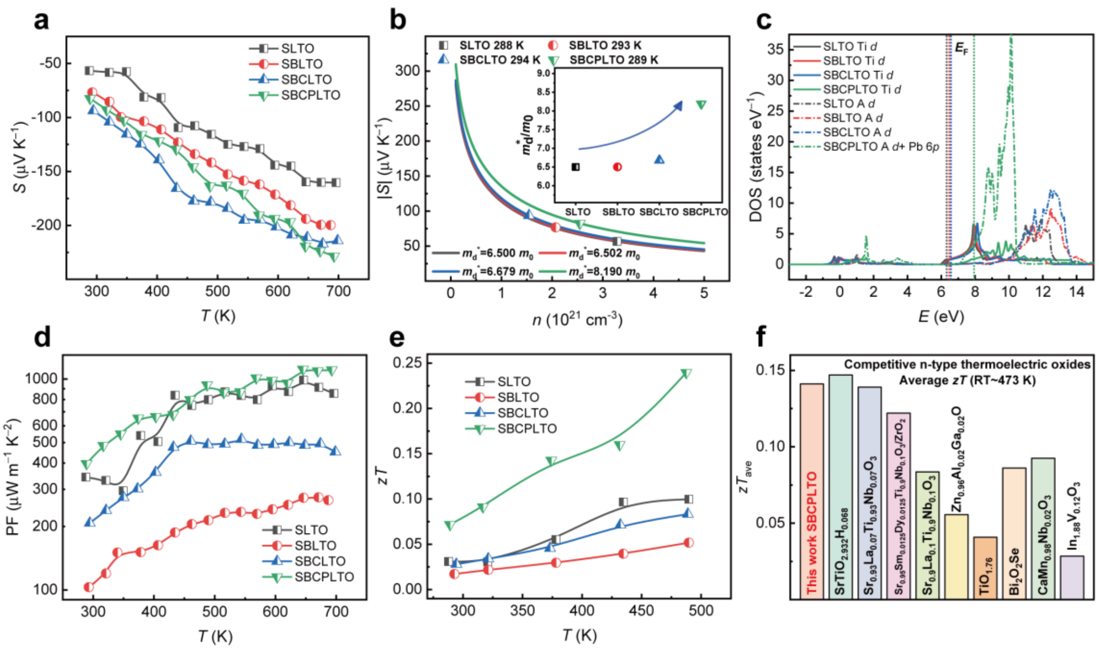

Supplementary Fig. 23 | The Seebeck related behaviors and thermoelectric performances. a. Temperature-dependent Seebeck coefficient. **b.** Pisarenko plot with an inset to display the enhancement of effective mass in SBCPLTO. The arrow is a guide for eyes. **c.** Partial density of states (DOS) of entropy engineered thin films. Only A-site *d*, Ti 3*d*, and Pb 6*p* orbitals were plotted for simplification. The atom of site A is Sr, La for SLTO; Sr, Ba, La for SBLTO; Sr, Ba, Ca, La for SBCLTO; Sr, Ba, Ca, Pb, La for SBCPLTO. The Fermi level is marked by dashed line in the same color with curves of corresponding composition. **d.-e.** Temperature-dependent power factors (PF, in log scale) (**d.**), zT (**e.**) of entropy engineered thin films. **f.** Average zT_{ave} (RT~473 K) comparison among SBCPLTO and other competitive *n*-type thermoelectric pure oxides[17,18,19,20,21,22,23–25].

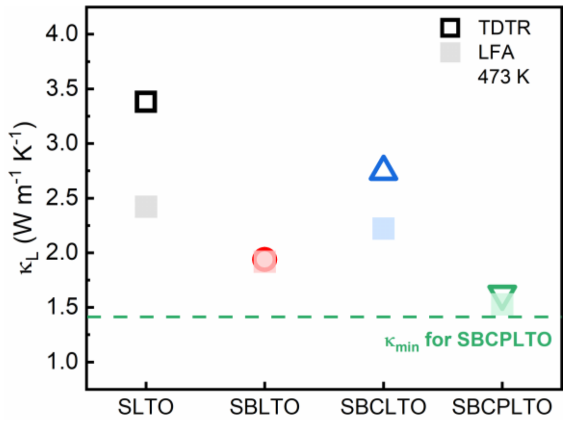

Supplementary Fig. 24 | The lattice thermal conductivity of SLTO, SBLTO, SBCLTO, SBCPLTO and corresponding unannealed bulks at 473 K for comparison. The minimum thermal conductivity of SBCPLTO was plotted in green dashed line for reference.

Notes for Supplementary Fig. 24:

The thermal conductivity of the corresponding unannealed bulks was also measured by LFA for reference and certainty, considering the limit of TDTR measurement including the measuring directions, measuring temperature.

The lattice thermal conductivity κ_L (SLTO)>κ_L (SBCLTO)>κ_L (SBLTO)>κ_L (SBCPLTO) were both found in films and the corresponding unannealed bulks (Supplementary Fig. 24), and results were close between LFA and TDTR, bulks and films. It was speculated that the anisotropy of the films and the interface scattering were not obvious due to their rather large thickness compared to l_p, relaxed strain (according to RSM results in Supplementary Fig. 4), and pseudo-cubic nature. It is worth noting that the thermal capacity was not necessarily monotonically correlated with entropy, and the Ca with smaller mass could lead to larger thermal capacity, thus larger thermal conductivity in SBCLTO than that in SBLTO (Fig. 1a-1b), however l_p is mainly decided by scattering, thus reversely-correlated to increased entropy (Supplementary Table 7).

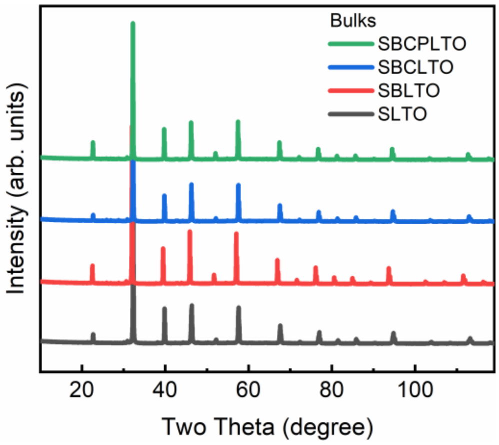

Supplementary Fig. 25 | The XRD of corresponding unannealed bulks of single phase.

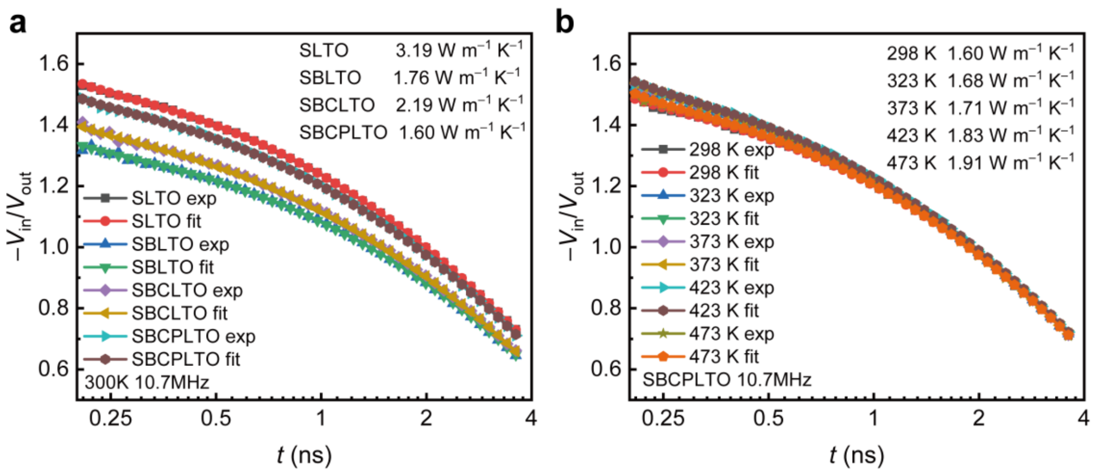

Supplementary Fig. 26 | The TDTR experimental data (10.7 MHz) and fitting curves. a. TDTR of SLTO, SBLTO, SBCLTO and SBCPLTO at 300 K. **b.** TDTR of SBCPLTO at different temperatures.

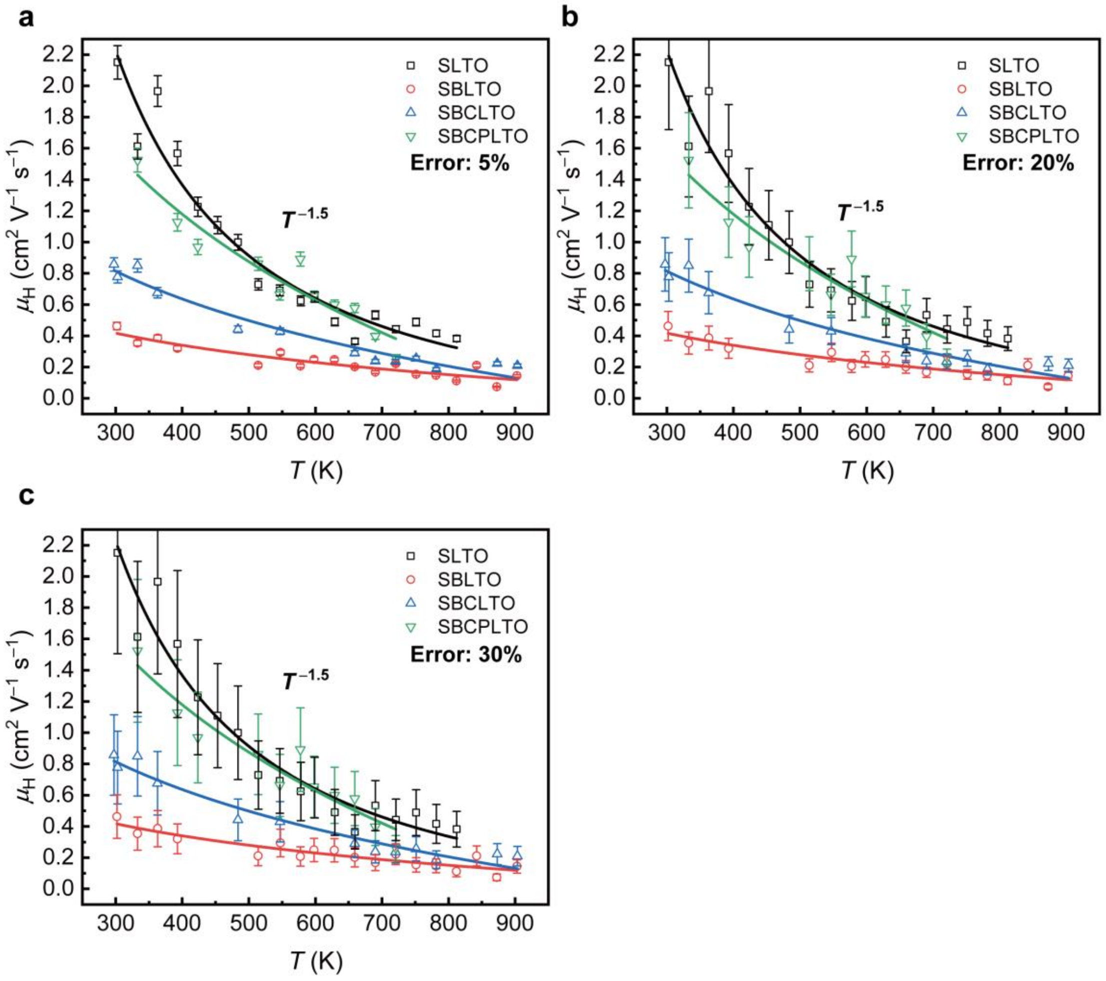

Supplementary Fig. 27 | The temperature variant Hall mobility with error bar. a. 5% (calculated error) error bar. **b.** and **c.** Exaggerated error bar with error 20% (**b.**) and 30% (**c.**).

Notes for Supplementary Fig. 27:

It has long been a difficult point to measure the Hall voltage of thermoelectric oxides with high carrier concentration (~10^{21} cm^{-3}) and low carrier mobility (~10^{-1}-10^{0} cm^2 V^{-1} s^{-1}), for the Hall voltage and Hall coefficient ($V_H=IB/ned$, $R_H=1/ne$) in this case could be too small to detect, and any disturbance during measurement could significantly affect the results of Hall voltage[26]. In this work, the mobility of samples varies from 0.1-2.2 cm^2 V^{-1} s^{-1}, right at the test limit of DC field measurement (1 to 1×10^6 cm^2 V^{-1} s^{-1}). Therefore, the authors decided to use the AC field measurement developed by the Lake Shore Cryotronics[27,28], using AC magnetic field rather than the traditional DC mode, to remove the

effect of misalignment, to enlarge the measure range of Hall mobility (1×10^{-3} to 1×10^{6} cm^2 V^{-1} s^{-1}) and to make the measurement more precise. After the application of AC field measurement, the Hall measurement could be performed and reasonable data could be obtained.

In terms of error bar (standard deviation), according to the manual given by Lake Shore official website, "all field values are nominal and can vary ±1%", and $\mu_H=V_H d\sigma/IB$, $\sigma=I'l/U'ad$, the error of μ_H could be calculated by the sum of the error (1%) of five field-related variants (V_H, I, B, I', U') to be 5%. The μ_H -T correlation with error of 5% was plotted in Supplementary Fig. 27. As can be seen in Supplementary Fig. 27a, the 5% error bar is not notable. However, multiple factors could enlarge the error of Hall measurement, like measuring geometry, electrical contact, shape and size of metal pads, etc., and the error could even reach 20%. The authors also plotted μ_H -T correlation with error of 20%, and 30% to exaggerate in Supplementary Fig. 27b and Supplementary Fig. 27c. As can be seen in Supplementary Fig. 27, the μ_H differences between samples are still more significant than the error bar.

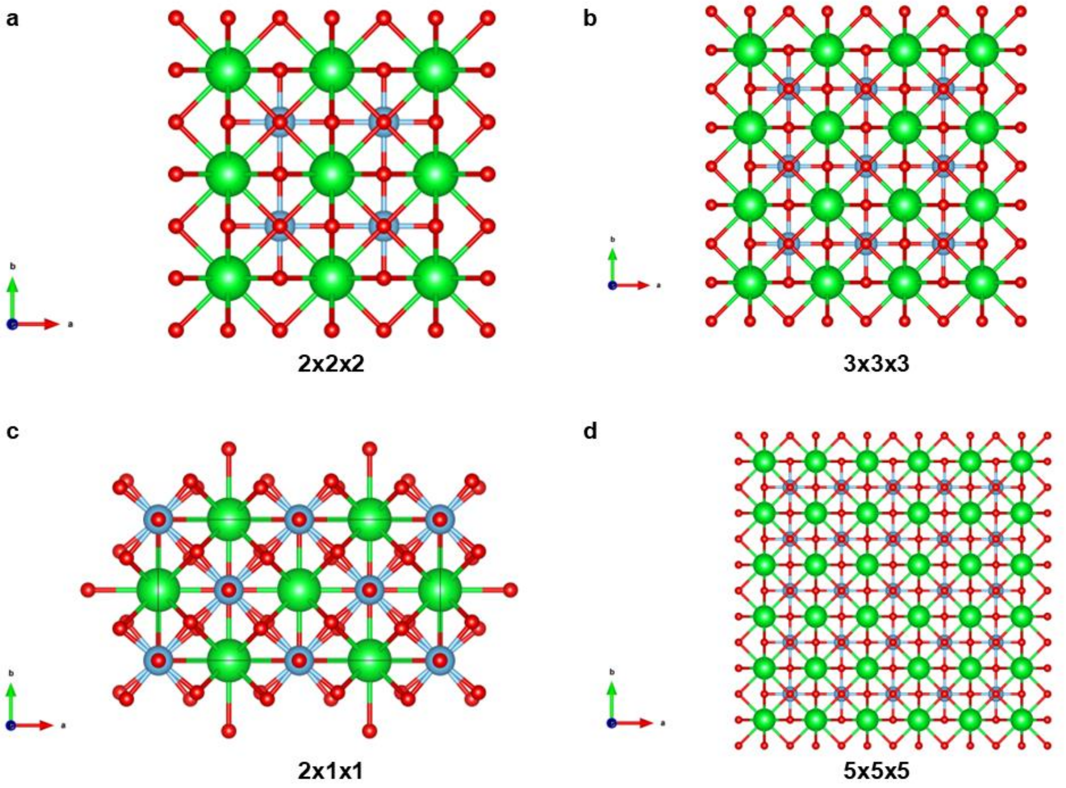

Supplementary Fig. 28 | $SrTiO_3$ supercell used for deep learning potential model as approximation. a. cubic 2×2×2; **b.** cubic 3×3×3; **c.** tetragonal 3×3×3; **d.** 5×5×5.

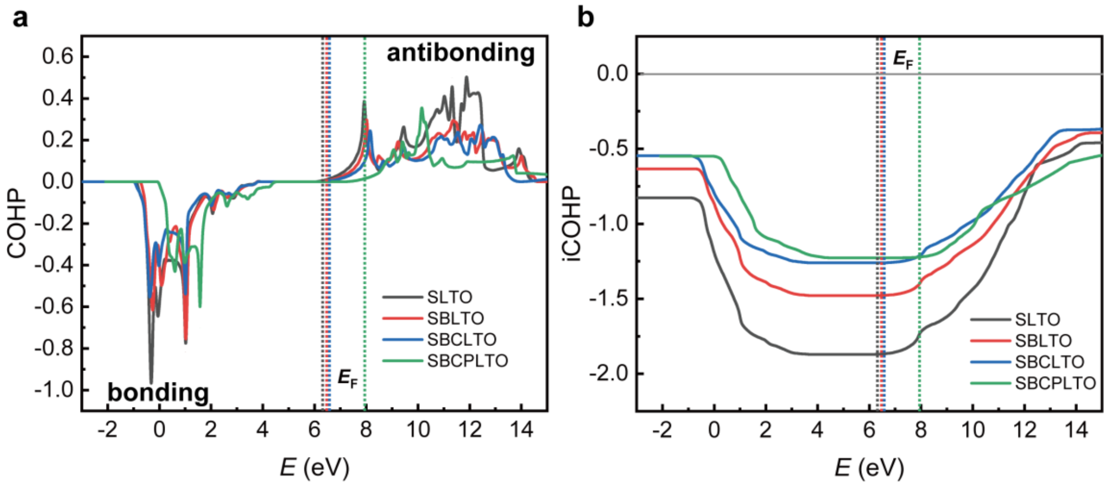

Supplementary Fig. 29 | COHP calculation. a. COHP calculation; **b.** integrated iCOHP calculation. The Fermi level is marked by dashed line in the same color with curves of corresponding composition.

Notes for Supplementary Fig. 29:

From the COHP result in Supplementary Fig. 29, the negative part of COHP below Fermi level represents bonding states, and the positive part over Fermi level represents antibonding states. Like the results got from DOS calculation, the antibonding and bonding states near the Fermi level mainly contribute to the Ti-O antibonding and bonding in SLTO, SBLTO, SBCLTO, and the A-side *d* orbitals, Ti *d* orbitals and Pb 6*p* orbitals participates in the states near the Fermi level in SBCPLTO. It could be speculated that the density of states of SBCPLTO is enhanced from the higher COHP at Fermi level. And since the iCOHP SLTO<SBLTO<SBCLTO<SBCPLTO<0 at Fermi level, the composition is thermodynamically stable, and the bonds of SBCPLTO is estimated to be more soft than other bonds. Limited by the VCA method, the COHP could only be taken as reference for trends.

Supplementary Tables

Supplementary Table 1 | The value of entropy calculated according to the nominal composition without taking Pb vacancies and O vacancies into account.

Nominal composition	Entropy (*R*)
$(Sr_{0.8}La_{0.2})TiO_3$	0.50
$(Sr_{0.4}Ba_{0.4}La_{0.2})TiO_3$	1.05
$Sr_{0.267}Ba_{0.267}Ca_{0.267}La_{0.2})TiO_3$	1.38
$(Sr_{0.2}Ba_{0.2}Ca_{0.2}Pb_{0.2}La_{0.2})TiO_3$	1.61

Supplementary Table 2 | The Shannon ionic radius and the molar mass of elements used in the entropy engineered perovskites[29].

Ion	Ionic Radius (Å)	Charge	Coordination	Molar mass
Sr	1.44	+2	12	87.62
Ba	1.61	+2	12	137.33
Ca	1.34	+2	12	40.08
Pb	1.49	+2	12	207.20
La	1.36	+3	12	138.91
Ti	0.605	+4	6	47.87
O	1.35	-2	2	16.00

Supplementary Table 3 | Quantitative element analyses of SBCPLTO surface by EPMA

Element	atom %	atom%/Ti%
Ti	5.0608	1.000
Ba	1.0162	0.201
Ca	1.2858	0.254
Pb	0.3687	0.073

Notes for Supplementary Table 3:

For SBCPLTO shared the same elements of Sr, La, O, and the LSAT substrates had strong signal of Sr, La, O, Al, Ta due to the depth of signal. Only results of Ti, Ba, Ca, Pb were listed in Supplementary Table 3, and the ratio to Ti was applied to represent the element concentration in the thin films. The Ba and Ca followed the designed composition, while the volatile Pb and Pb vacancies occupied A-sites by 7.3% (Pb/Ti) and 12.7% (V_{Pb}/Ti, nominal).

Supplementary Table 4 | The mean radius $\bar{r}$ at A sites and the size disorder parameter δ at A sites of entropy engineered thin films[12].

Nominal composition	$\bar{r}$ (Å)	δ
$(Sr_{0.8}La_{0.2})TiO_3$	1.42	0.0225
$(Sr_{0.4}Ba_{0.4}La_{0.2})TiO_3$	1.49	0.0675
$Sr_{0.267}Ba_{0.267}Ca_{0.267}La_{0.2})TiO_3$	1.44	0.0748
$(Sr_{0.2}Ba_{0.2}Ca_{0.2}Pb_{0.2}La_{0.2})TiO_3$	1.45	0.0673

Notes for Supplementary Table 4:

It was difficult to estimate the radius of Pb vacancies, and the amounts of the Pb vacancies in the films were also hard to be measured accurately, even EPMA could give quantitative reference. Considering that, the size disorder parameter was estimated without taking Pb vacancies into account. It is reasonable to speculate that the mean radius $\bar{r}$ would decrease and the size disorder parameter δ would increase for SBCPLTO, which would be more consistent with the phonon MFP l_p-δ correlation in Fig. 1c. The definition of the mean radius $\bar{r}$ and the size disorder parameter δ were described in the supplementary materials Supplementary Equation 1 and Supplementary Equation 2.

Supplementary Table 5 | The quantitative elemental analysis of SBCPLTO corresponding bulks by EDS.

Elements	Atomic %	Atomic %/Ti %
Ti	22.51	1.000
O	54.69	2.430
Ca	4.25	0.189
Sr	4.85	0.215
Ba	5.6	0.249
La	5.2	0.231
Pb	2.9	0.129

Notes for Supplementary Table 5:

The value of Pb/Ti ~0.129 in corresponding bulks deviated from designed 0.2 due to the volatilization of Pb during the calcination and sintering. After film growth and annealing, more Pb vacancies formed, and the Pb/Ti value was 0.073 in the SBCPLTO films (Supplementary Table 3). The bulks could give structural and transport reference, considering that the effects from Pb vacancies could already be reflected by the large Pb/Ti deviation from designed 0.2.

Supplementary Table 6 | The average bond length and the deduced tolerance factor *t* obtained by fitting the synchrotron radiation X-ray scattering PDF on corresponding bulks at room temperature.

Samples	Length (La-O) Å	Length (Ti-O) Å	t_{obs}
SLTO	2.7835	1.9855	0.9913
SBLTO	2.7713	1.9962	0.9817
SBCLTO	2.7524	1.9858	0.9801
SBCPLTO	2.7692	1.9875	0.9853

Supplementary Table 7 | The parameters of phonon transport in corresponding bulks at room temperature.

Samples	δ	v_l	v_t	v_a	κ_L	C_V	l_p
		m s^{-1}	m s^{-1}	m s^{-1}	W m^{-1} K^{-1}	J cm^{-3} K^{-1}	Å
SLTO	0.022	4775.7	2040.9	2306.6	2.89	2.96	12.70
SBLTO	0.067	6957.3	3969.0	4410.9	2.22	2.19	6.89
SBCLTO	0.075	7218.2	3981.9	4437.3	2.64	3.09	5.78
SBCPLTO	0.067	6714.0	3690.1	4113.3	1.80	2.38	5.52

Supplementary Table 8 | The parameters of carrier transport in thin films at room temperature.

Samples	t	n	μ_H	μ_w	m_I^*	v_l	ρ	B_E	Ξ_{def}	l_c
		10^{21} cm^{-3}	cm^2 V^{-1} s^{-1}		10^{-30} kg	m s^{-1}	g cm^{-3}	μW m^{-1} K^{-2}	eV	Å
SLTO	0.9913	3.28	2.15	78.59	1.74	4775.7	5.43	164.2	20.8	3.58
SBLTO	0.9817	2.07	0.46	17.85	1.74	6957.3	5.80	37.3	65.7	0.66
SBCLTO	0.9801	1.55	0.86	26.10	1.79	7218.2	5.39	54.6	53.6	1.12
SBCPLTO	0.9853	2.55	1.64	65.06	2.14	6714.0	5.96	136.8	30.2	2.51

Supplementary Table 9 | The comprehensive carrier-phonon transport parameters and correlated structural descriptor at room temperature.

Samples	t_{obs}	Ξ_{def}<br>eV	l_c<br>Å	δ	l_p<br>Å
SLTO	0.9913	20.8	3.58	0.022	12.70
SBLTO	0.9817	65.7	0.66	0.067	6.89
SBCLTO	0.9801	53.6	1.12	0.075	5.78
SBCPLTO	0.9853	30.2	2.51	0.067	5.52

Supplementary Table 10 | The states and types of the TiO_6 distortion of entropy engineered thin films.

	SLTO	SBLTO	SBCLTO	SBCPLTO
Tilting	Slight	Ignorable	Moderate	Ignorable
Displacement	Ignorable	Large	Moderate	Ignorable

Supplementary Table 11 | The average normalized Ti displacement $\bar{\boldsymbol{d}}_{\mathrm{Ti}}$ and the standard deviation $\boldsymbol{\delta_d}$.

	SLTO	SBLTO	SBCLTO	SBCPLTO
$\bar{\boldsymbol{d}}_{\mathrm{Ti}}$	0.0184	0.0900	0.0501	0.0115
$\boldsymbol{\delta_d}$	0.0067	0.0266	0.0214	0.0073

Reference

1. Wright, A. J. *et al.* Size disorder as a descriptor for predicting reduced thermal conductivity in medium- and high-entropy pyrochlore oxides. *Scr. Mater.* **181**, 76–81 (2020).
2. Ma, Z. *et al.* High Thermoelectric Performance and Low Lattice Thermal Conductivity in Lattice-Distorted High-Entropy Semiconductors AgMnSn1-xPbxSbTe4. *Chem. Mater.* **34**, 8959–8967 (2022).
3. Goldsmid, H. J. *Optimisation and Selection of Semiconductor Thermoelements*. *Springer Series in Materials Science* vol. 121 (Springer, 2016).
4. Snyder, G. J. *et al.* Weighted Mobility. *Adv. Mater.* **32**, 1–5 (2020).
5. Zhang, X. *et al.* Electronic quality factor for thermoelectrics. *Sci. Adv.* **6**, 6–11 (2020).
6. Cahill, D. G., Watson, S. K. & Pohl, R. O. Lower limit to the thermal conductivity of disordered crystals. *Phys. Rev. B* **46**, 6131–6140 (1992).
7. Cao, Y. *et al.* Unraveling the relationships between chemical bonding and thermoelectric properties: n-type ABO3 perovskites. *J. Mater. Chem. A* 121 (2022) doi:10.1039/d2ta01624a.
8. Su, L. *et al.* High thermoelectric performance realized through manipulating layered phonon-electron decoupling. *Science (80-.).* **375**, 1385–1389 (2022).
9. Fu, C. *et al.* Enhancing the Figure of Merit of Heavy-Band Thermoelectric Materials Through Hierarchical Phonon Scattering. *Adv. Sci.* **3**, 1–7 (2016).
10. Zhu, T., Gao, H., Chen, Y. & Zhao, X. Ioffe-Regel limit and lattice thermal

conductivity reduction of high performance (AgSbTe2)15(GeTe)85 thermoelectric materials. *J. Mater. Chem. A* **2**, 3251–3256 (2014).

11. Zheng, Y. *et al.* Electrical Property Enhancement in Orientation-Modulated Perovskite La-Doped SrTiO 3 Thermoelectric Thin Films. *Adv. Funct. Mater.* **33**, 1–11 (2023).

12. Marrocchelli, D., Bishop, S. R., Tuller, H. L. & Yildiz, B. Understanding chemical expansion in non-stoichiometric oxides: Ceria and zirconia case studies. *Adv. Funct. Mater.* **22**, 1958–1965 (2012).

13. Wu, R. *et al.* Strong charge carrier scattering at grain boundaries of PbTe caused by the collapse of metavalent bonding. *Nat. Commun.* **14**, 719 (2023).

14. Isotta, E. *et al.* Microscale Imaging of Thermal Conductivity Suppression at Grain Boundaries. *Adv. Mater.* **35**, 1–23 (2023).

15. Takizawa, M. *et al.* Angle-resolved photoemission study of Nb-doped SrTiO3 . *Phys. Rev. B* **79**, 1–4 (2009).

16. Alkathy, M. S., Zabotto, F. L., Milton, F. P. & Eiras, J. A. Bandgap tuning in samarium-modified bismuth titanate by site engineering using iron and cobalt co-doping for photovoltaic application. *J. Alloys Compd.* **908**, 164222 (2022).

17. Wang, J. *et al.* Record high thermoelectric performance in bulk SrTiO3 via nano-scale modulation doping. *Nano Energy* **35**, 387–395 (2017).

18. Ohtaki, M., Araki, K. & Yamamoto, K. High thermoelectric performance of dually doped ZnO ceramics. *J. Electron. Mater.* **38**, 1234–1238 (2009).

19. Ahmad, A. *et al.* Thermoelectric Performance Enhancement of Vanadium

Doped n-Type In2O3 Ceramics via Carrier Engineering and Phonon Suppression. *ACS Appl. Energy Mater.* **3**, 1552–1558 (2020).

20. Bocher, L. *et al.* CaMn1-x NbxO3 (x ≤ 0.08) perovskite-type phases as promising new high-temperature n-type thermoelectric materials. *Inorg. Chem.* **47**, 8077–8085 (2008).

21. Liu, H. *et al.* Enhanced thermoelectric properties of nonstoichiometric TiO1.76 with excellent mechanical properties induced by optimizing processing parameters. *Ceram. Int.* **44**, 19859–19865 (2018).

22. Tan, X. *et al.* Synergistical Enhancement of Thermoelectric Properties in n-Type Bi2O2Se by Carrier Engineering and Hierarchical Microstructure. *Adv. Energy Mater.* **9**, 1–7 (2019).

23. He, X. *et al.* Hydride Anion Substitution Boosts Thermoelectric Performance of Polycrystalline SrTiO3 via Simultaneous Realization of Reduced Thermal Conductivity and High Electronic Conductivity. *Adv. Funct. Mater.* **33**, (2023).

24. Bakhshi, H., Sarraf-Mamoory, R., Yourdkhani, A., Abdelnabi, A. A. & Mozharivskyj, Y. Highly dense Sr0.95Sm0.0125Dy0.0125□0.025Ti0.90Nb0.10O3±: δ/ZrO2 composite preparation directly through spark plasma sintering and its thermoelectric properties. *Dalt. Trans.* **49**, 17–22 (2019).

25. Li, J. B. *et al.* Broadening the temperature range for high thermoelectric performance of bulk polycrystalline strontium titanate by controlling the electronic transport properties. *J. Mater. Chem. C* **6**, 7594–7603 (2018).

26. Putley, E. H. & Rice, S. A. The Hall Effect and Related Phenomena . *Phys. Today* **15**, 72–72 (1962).

27. Lindermuth, D. J. R. An introduction to AC field Hall effect measurements. *J. Cryst. Growth* **36**, 29–35 (1976).

28. Lindemuth, J., Dodrill, B., Meyer, J. & Vurgaftman, I. Extraction of Low Mobility , Low Conductivity Carriers from Field. 48–50 (2002).

29. Shannon, R. D. Revised effective ionic radii and systematic studies of interatomic distances in halides and chalcogenides. *Acta Crystallogr. Sect. A* **32**, 751–767 (1976).
