# Supplementary material for: Carrier-phonon decoupling in perovskite thermoelectrics via entropy engineering
Source: Nat Commun. 2024 Sep 3;15:7650. doi: 10.1038/s41467-024-52063-5 (PMC11369264; doi:10.1038/s41467-024-52063-5)
Supplement: Supplementary file 3 — Description of Additional Supplementary Files [file 41467_2024_52063_MOESM3_ESM.pdf]

Description of Additional Supplementary Files

Supplementary Data 1| 2×1×1 $SrTiO_3$ tetragonal supercell for deep learning potential model

Supplementary Data 2| 2×2×2 $SrTiO_3$ cubic supercell for deep learning potential model

Supplementary Data 3| 3×3×3 $SrTiO_3$ cubic supercell for deep learning potential model

Supplementary Data 4| 5×5×5 SrTiO3 cubic supercell

Supplementary Data 5| $SrTiO_3$ structure generated for computing phonon spectrum

Supplementary Data 6| $(Sr_{0.8}La_{0.2})TiO_3$ structure generated for computing phonon spectrum

Supplementary Data 7|$(Sr_{0.4}Ba_{0.4}La_{0.2})TiO_3$ structure generated for computing phonon spectrum

Supplementary Data 8|$(Sr_{0.27}Ba_{0.27}Ca_{0.27}La_{0.2})TiO_3$ structure generated for computing phonon spectrum

Supplementary Data 9|$(Sr_{0.2}Ba_{0.2}Ca_{0.2}Pb_{0.2}La_{0.2})TiO_3$ structure generated for computing phonon spectrum
