# Supplementary material for: Carrier-phonon decoupling in perovskite thermoelectrics via entropy engineering
Source: Nat Commun. 2024 Sep 3;15:7650. doi: 10.1038/s41467-024-52063-5 (PMC11369264; doi:10.1038/s41467-024-52063-5)
Supplement: Supplementary file 14 — Reporting Summary [file 41467_2024_52063_MOESM14_ESM.pdf]

nature portfolio

Corresponding author(s): Yuan-Hua Lin

Last updated by author(s): 2024-08-19

Reporting Summary

Nature Portfolio wishes to improve the reproducibility of the work that we publish. This form provides structure for consistency and transparency in reporting. For further information on Nature Portfolio policies, see our Editorial Policies and the Editorial Policy Checklist.

Statistics

For all statistical analyses, confirm that the following items are present in the figure legend, table legend, main text, or Methods section.

n/a	Confirmed	
☐	☒	The exact sample size (n) for each experimental group/condition, given as a discrete number and unit of measurement
☐	☒	A statement on whether measurements were taken from distinct samples or whether the same sample was measured repeatedly
☐	☒	The statistical test(s) used AND whether they are one- or two-sided<br>*Only common tests should be described solely by name; describe more complex techniques in the Methods section.*
☒	☐	A description of all covariates tested
☒	☐	A description of any assumptions or corrections, such as tests of normality and adjustment for multiple comparisons
☐	☒	A full description of the statistical parameters including central tendency (e.g. means) or other basic estimates (e.g. regression coefficient) AND variation (e.g. standard deviation) or associated estimates of uncertainty (e.g. confidence intervals)
☒	☐	For null hypothesis testing, the test statistic (e.g. F, t, r) with confidence intervals, effect sizes, degrees of freedom and P value noted<br>*Give P values as exact values whenever suitable.*
☐	☒	For Bayesian analysis, information on the choice of priors and Markov chain Monte Carlo settings
☐	☒	For hierarchical and complex designs, identification of the appropriate level for tests and full reporting of outcomes
☒	☐	Estimates of effect sizes (e.g. Cohen's d, Pearson's r), indicating how they were calculated

Our web collection on statistics for biologists contains articles on many of the points above.

Software and code

Policy information about availability of computer code

Data collection	OR
Data analysis	OR

For manuscripts utilizing custom algorithms or software that are central to the research but not yet described in published literature, software must be made available to editors and reviewers. We strongly encourage code deposition in a community repository (e.g. GitHub). See the Nature Portfolio guidelines for submitting code & software for further information.

Data

Policy information about availability of data

All manuscripts must include a data availability statement. This statement should provide the following information, where applicable:

- Accession codes, unique identifiers, or web links for publicly available datasets
- A description of any restrictions on data availability
- For clinical datasets or third party data, please ensure that the statement adheres to our policy

The authors declare that the data supporting the findings of this study are available within the paper and its Supplementary Information files. All of the other data are available from the authors upon reasonable request.

Human research participants

Policy information about studies involving human research participants and Sex and Gender in Research.

Reporting on sex and gender	This information has not been collected.
Population characteristics	See above.
Recruitment	OR
Ethics oversight	OR

Note that full information on the approval of the study protocol must also be provided in the manuscript.

Field-specific reporting

Please select the one below that is the best fit for your research. If you are not sure, read the appropriate sections before making your selection.

☐ Life sciences ☐ Behavioural & social sciences ☒ Ecological, evolutionary & environmental sciences

For a reference copy of the document with all sections, see nature.com/documents/nr-reporting-summary-flat.pdf

Ecological, evolutionary & environmental sciences study design

All studies must disclose on these points even when the disclosure is negative.

Study description	Here, we propose an entropy engineering strategy to realize the carrier-phonon decoupling in the typical SrTiO3-based perovskite thermoelectrics. By high-entropy design, the lattice thermal conductivity could be reduced nearly to the amorphous limit, 1.25 W m-1 K-1. Simultaneously, entropy engineering can tune the Ti displacement, improving the weighted mobility to 65 cm2 V-1 s-1. Such carrier-phonon decoupling behaviors enable the greatly enhanced μW/κL of ~5.2×103 cm3 K J-1 V-1. The measured maximum zT of 0.24 at 488 K and the estimated zT of ~0.8 at 1173 K in (Sr0.2Ba0.2Ca0.2Pb0.2La0.2)TiO3 film are among the best of n-type thermoelectric oxides. These results reveal that the entropy engineering may be a promising strategy to decouple the carrier-phonon transport and achieve higher zT in thermoelectrics.
Research sample	SrTiO3-based thin films
Sampling strategy	The samples were made according to our design.
Data collection	The data were collected by devices.
Timing and spatial scale	The data were collected under the same condition.
Data exclusions	No data were excluded from the analyses.
Reproducibility	All attempts to repeat the experiment were successful.
Randomization	Not relevant to our study since the samples were made according to our design.
Blinding	Not relevant to our study since the samples were made according to our design.

Did the study involve field work? ☐ Yes ☒ No

Reporting for specific materials, systems and methods

We require information from authors about some types of materials, experimental systems and methods used in many studies. Here, indicate whether each material, system or method listed is relevant to your study. If you are not sure if a list item applies to your research, read the appropriate section before selecting a response.

Materials & experimental systems

n/a	Involved in the study	
☒	☐	Antibodies
☒	☐	Eukaryotic cell lines
☒	☐	Palaeontology and archaeology
☒	☐	Animals and other organisms
☒	☐	Clinical data
☒	☐	Dual use research of concern

Methods

n/a	Involved in the study	
☒	☐	ChIP-seq
☒	☐	Flow cytometry
☒	☐	MRI-based neuroimaging
